# Transanal Drainage of Coloanal Anastomotic Leaks

**DOI:** 10.1155/2018/9806259

**Published:** 2018-03-27

**Authors:** Bradley Sherman, Mark Arnold, Syed Husain

**Affiliations:** ^1^Department of Surgery, OhioHealth Doctors Hospital, 5100 West Broad Street, Columbus, OH 43228, USA; ^2^Division of Colon and Rectal Surgery, Wexner Medical Center, The Ohio State University, N737 Doan Hall, 410 West 10th Avenue, Columbus, OH 43210, USA

## Abstract

The conventional operative intervention for leaks following coloanal anastomoses has been proximal fecal diversion with or without take-down of anastomosis. A few of these cases are also amenable to percutaneous drainage. Ostomies created in this situation are often permanent, specifically in cases where coloanal anastomoses are taken down at the time of reoperation. We present two patients who developed perianastomotic pelvic abscesses that were treated with transanal large bore catheter drainage resulting in successful salvage of coloanal anastomoses without the need for a laparotomy or ostomy creation. We propose this to be an effective therapeutic approach to leaks involving low coloanal anastomoses in the absence of generalized peritonitis.

## 1. Introduction

Anastomotic leaks carry a reported mortality of 6 to 39% [1]. Majority of these patients require a reoperation with complete take-down of anastomosis and fecal diversion. Take-down of coloanal anastomosis presents a unique challenge as pelvic scarring, and absence of distal bowel segment precludes restoration of bowel continuity at a later day.

We present two patients managed with a transanal drainage following coloanal anastomotic dehiscence.

## 2. Case 1

An 86-year-old female underwent uncomplicated Altemeier procedure for rectal prolapse and was discharged on postoperative day (POD) 5. She presented to the emergency department two days after her discharge with fevers up to 102.6°F, pelvic pain, fatigue, and anorexia.

On exam, she was febrile to 101.6°F, pulse was 79, and blood pressure was 117/58. Her abdominal exam did not reveal any evidence of generalized peritonitis, and lower abdominal tenderness was present. Her WBC count was 13.1. Imaging demonstrated a large fluid collection and gas within the pelvis that tracked cephalad in the retroperitoneum.

She was admitted, made NPO, and started on antibiotics. Interventional radiology deemed the abscess not amenable to percutaneous drainage. Thus, we proceeded with an anorectal examination under anesthesia for surgical drainage of the area and stoma creation, if needed. She was noted to have a grossly intact anastomosis. A vaginal exam demonstrated fluctuant swelling in the posterior vaginal vault, and a 18-gauge needle was used to aspirate 30 mL of clear straw-colored fluid and air. A diagnostic laparoscopy was then performed demonstrating normal-appearing bowel and pelvis without evidence of fecal matter. We felt that the collection represented a post-op seroma, and operation was concluded at this time.

**Figure 1:**
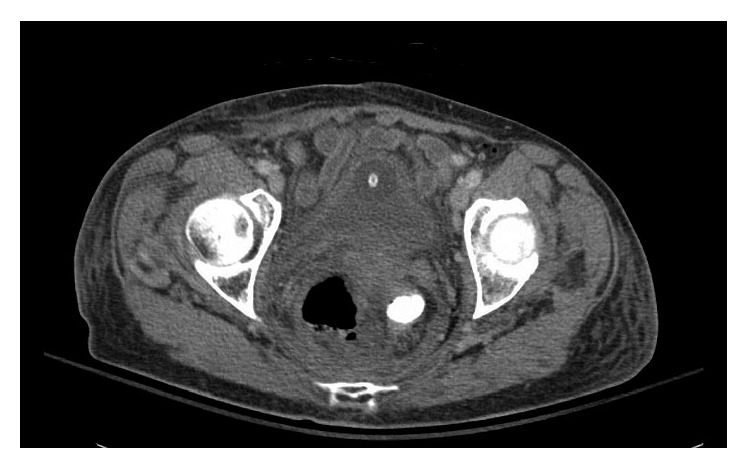
Perirectal fluid collection with contrast in the rectal lumen.

The patient was maintained on empiric IV antibiotics, and her diet was resumed. She remained hemodynamically stable however continued to experience ongoing fevers. A CT scan was repeated on hospital day 6 (Figure 1), demonstrating an enlarging fluid collection containing enteric contrast measuring 5.8 × 7.5 cm, previously 5.4 × 6.4 cm.

Once again, interventional radiology determined that the collection was not suitable for percutaneous approach, and she was taken back to the operating room. We were unsuccessful in accessing the abscess cavity via a skin incision on the right lateral aspect of coccyx. An anorectal exam under anesthesia revealed anastomotic defect extending about 1.5 proximally. The abscess cavity was suctioned out via this defect, and a 26-French Malecot drain was placed in the cavity transanally. The drain was then secured to perianal skin via two nylon sutures.

**Figure 2:**
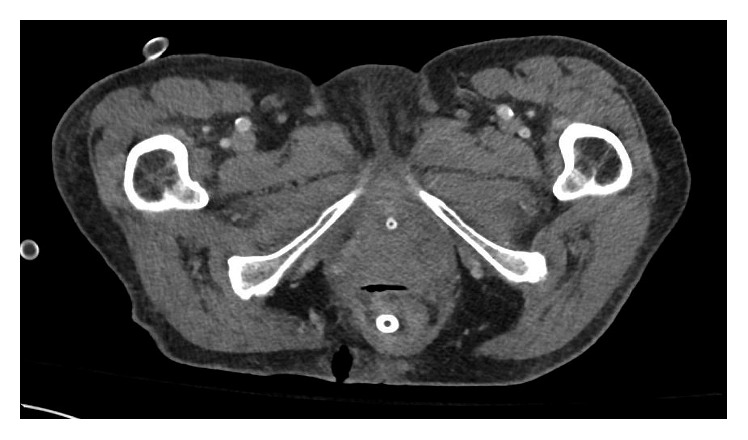
CT on day 7 after drainage demonstrating resolution of abscess with transanal drain in place.

**Figure 3:**
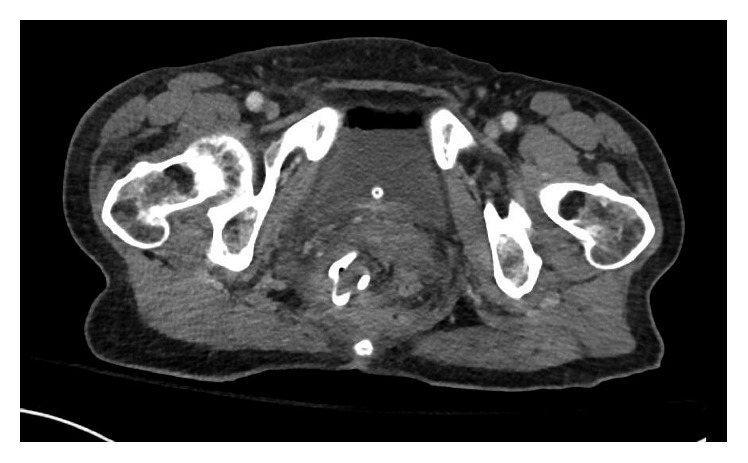
CT on day 15 after drainage demonstrating resolution of abscess with transanal drain in place.

Repeat CT scans done on 7 and 15 days after the drainage demonstrated resolving fluid collections (Figures 2 and 3). The patient did well and was discharged on hospital day 19 with a plan to maintain the drain for 4–6 weeks to allow complete collapse of abscess cavity.

The patient presented again two weeks later after spontaneous removal of Malecot drain. An exam under anesthesia was performed revealing involution of abscess cavity into fibrotic sinus tract. This tract was incorporated into the bowel lumen by dividing the septum with LigaSure device precluding the possibility of recurrent abscess caused by the presence of a narrow sinus tract.

The patient was seen in follow-up in the office two months after her last procedure. She reported minimal residual rectal discomfort, was continent without recurrent prolapse, and endorsed normal bowel function.

## 3. Case 2

A 61-year-old male underwent low anterior resection for rectal cancer. He was discharged on postoperative day 11 after recovering from post-op ileus. Six days later, he presented to the emergency department with acute onset of abdominal pain. He was afebrile, had normal heart rate, and was mildly hypertensive on presentation. Although diffuse abdominal tenderness was present, there were no signs of peritonitis on exam. WBC count was 11.8K. CT scan demonstrated possible dehiscence at the anastomotic site and presence of enteric contrast in a pelvic collection measuring 4 × 5.1 cm without gross contamination of peritoneal cavity.

He was taken to the operating room as the abscess was determined inaccessible via percutaneous route. The exam revealed a small posterior disruption of coloanal anastomosis. The cavity was suctioned out, and a transanal 26-French Malecot drain was placed.

**Figure 4:**
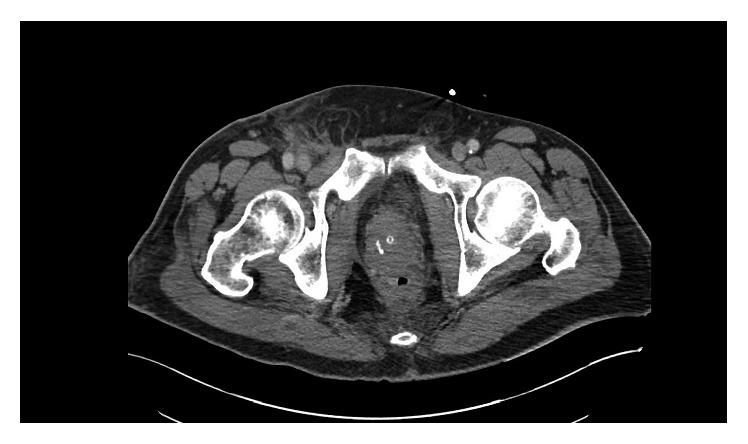
Postdrainage resolution of perirectal collection.

Diet was resumed a week after drainage, and drain was removed prior to his discharge on day 10. Follow-up CT scan demonstrated near resolution of the previously visualized fluid collection and a smaller cavity that communicated with the rectum (Figure 4). The patient was seen in follow-up about four months after his last surgery and reported a complete recovery.

## 4. Discussion

Abdominal wash out with stoma creation is the treatment of choice for an anastomotic dehiscence in presence of peritonitis. Many of these patients also require a complete take-down of anastomosis [2]. The reported healing rates after diversion are widely variable, and this approach obviously subjects the patient to another abdominal surgery [3]. Contrary to leaks presenting with peritonitis, anastomotic dehiscence leading to localized peritoneal contamination and walled off abscess can often be treated with intravenous antibiotics with or without placement of interventional radiology-guided percutaneous drains.

Restoration of bowel continuity may not be possible after take-down of coloanal anastomosis; however, these leaks carry the benefit of accessibility via transanal route, which in select cases may allow for preservation of the anastomosis.

It is imperative to note that both of our cases were hemodynamically stable and did not exhibit any signs of generalized peritonitis. Furthermore, anastomotic failures encompassed a small portion of anastomotic circumference. We strongly caution against employment of this technique in patients with frank peritonitis, hemodynamic instability, or major anastomotic disruption which, in our opinion, are best managed with fecal diversion and anastomotic take-down.

Several previous publications have addressed the issue of anastomotic preservation with or without proximal diversion. Out of the techniques described, perhaps the most similar to our proposed method is the vacuum-assisted closure anastomotic leaks. Weidenhagen et al. [4] reported a success rate of 28 out of 29 patients treated with endoscopic suction-assisted drainage of anastomotic leaks after low anterior resections. Contrary to our patients, overwhelming majority of patients in this cohort had proximal fecal diversion, and only four patients were managed without a stoma. Other authors have described similar success rates with this technique; however, common to these reports is the need for frequent, multiple procedures due to the need for repeated debridements and sponge replacements [5, 6]. Verlaan et al. [7] reported successful management of five out of six colorectal anastomotic leaks using a modification of this technique combining endosponge placement with closure of anastomotic defect using sutures or an endoscopic clip. Finally, Gardenbroek et al. [8] compared endoscopic vacuum closure to conventional treatment for management of ileoanal pouch leaks. The authors reported similar results and concluded that vacuum-assisted closure of anastomotic leaks was a highly effective method of managing iloeanal pouch anastomotic leaks. While the above-described reports testify the effectiveness of suction-assisted closure of low anastomotic leaks, we feel that our proposed method offers a simple, low-cost alternative.

Possible complications of proposed approach include anal pain, poor bowel function, and compromised fecal continence. Many patients go on to develop a chronic sinus that could lead to fistula formation [9] as witnessed in one of our patients.

Our case report demonstrates that transanal drainage is a viable option that allows anastomotic preservation while avoiding fecal diversion in selected patients with low anastomotic leaks.
